# Successful surgical management of a giant floating aortic thrombus causing acute upper limb ischaemia: a case report

**DOI:** 10.1093/ehjcr/ytag270

**Published:** 2026-05-11

**Authors:** Mani Moayerifar, Behrad Eftekhari, Anita Khalili, Serban Stoica, Mahboobeh Gholipour

**Affiliations:** Healthy Heart Research Center, Heshmat Hospital, School of Medicine, Guilan University of Medical Sciences, Rasht, Iran; Department of Cardiac Surgery, Imam Khomeini Hospital Complex, Tehran University of Medical Sciences, Tehran, Iran; Department of Medicine, Guilan University of Medical Sciences, Rasht, Iran; Department of Medicine, Guilan University of Medical Sciences, Rasht, Iran; Department of Cardiac Surgery, University of Bristol, Bristol BS8 1QU, UK; Healthy Heart Research Center, Heshmat Hospital, School of Medicine, Guilan University of Medical Sciences, Rasht, Iran

**Keywords:** Aortic thrombus, Acute limb ischaemia, Mobile aortic thrombus, Surgical thrombectomy, Case report

## Abstract

**Background:**

Aortic thrombi are rare and pose clinical challenges as they often remain undetected until embolic complications occur.

**Case summary:**

We present the case of a 43-year-old male with acute limb ischaemia, revealed to be due to a large mobile thrombus (measuring 53 × 13 mm) extending from the ascending to descending aorta. The obstruction in the peripheral arteries was managed through mechanical thrombectomy, while the intra-aortic thrombus required surgical intervention. Following these procedures, the patient was put on Apixaban, and no signs of recurrence appeared in subsequent follow-up assessments.

**Discussion:**

Timely diagnosis of aortic thrombi is essential to prevent embolic events. Large floating thrombi are rare in healthy young individuals with no pre-existing conditions, especially when causing only upper limb ischaemia. Prompt surgical intervention is recommended to treat and prevent recurrence.

**Conclusion:**

While various strategies exist for dealing with large floating thrombi, prioritizing surgical intervention is essential to prevent potential future recurrences.

Learning points

- Mobile aortic mural thrombus can cause acute limb ischaemia in young patients with a normal, non-atherosclerotic aorta and requires prompt evaluation when cardiac workup is unremarkable.
- Early surgical excision is the preferred treatment for large, highly mobile aortic thrombi associated with embolic events, as anticoagulation alone carries a risk of recurrent embolism.

## Introduction

Floating aortic mural thrombi in an otherwise normal (non-aneurysmal, non-atherosclerotic) aorta are rare.^1,2^ These lesions are mobile and highly emboligenic, often leading to acute ischaemic events such as limb or visceral infarction.^2,3^ They usually occur in the setting of atherosclerosis or aneurysm, so their appearance in young healthy patients is particularly unusual.^2,4^ We report a 43-year-old man with acute upper limb ischaemia caused by a giant free-floating thrombus in the thoracic aorta, highlighting the importance of recognizing this rare entity in contemporary cardiovascular practice.

## Summary figure

**Figure:**
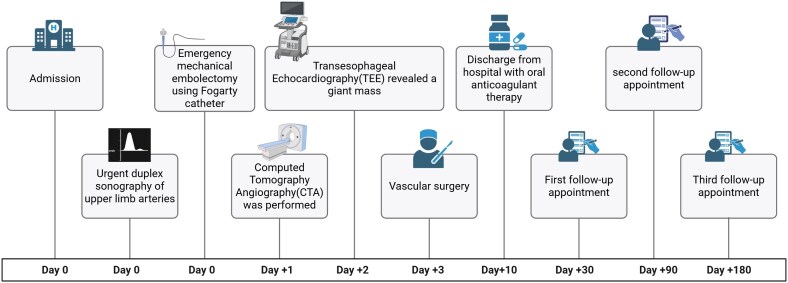


## Case presentation

**Figure 1:**
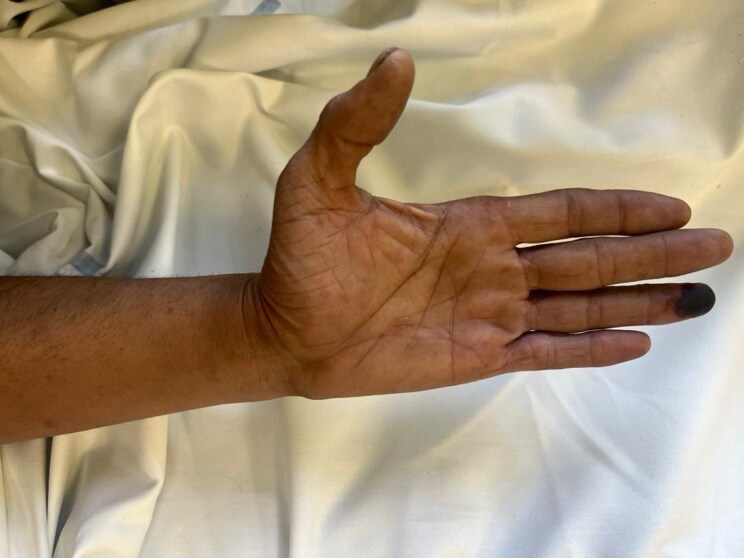
Patient's left hand upon admission.

A 43-year-old male presented to the emergency department with complaints of pain and a bluish-black discolouration at the tip of his left hand's fourth finger (*Figure 1*). The patient reported the sudden onset of pain, coldness, and paraesthesia in the affected area a few hours before hospital admission, indicative of acute limb ischaemia (ALI), Rutherford category IIb. Clinical examination revealed diminished radial pulse on the left compared to the right, with a significant discrepancy in blood pressure readings between the arms (left arm at 70 mm Hg, right arm at 140/80 mm Hg). The patient exhibited symptoms of acute upper limb ischaemia, including cyanosis and hypothermia of the hand and forearm, but no motor impairment was detected. The cardiopulmonary assessment was normal, including clear lung fields upon auscultation, typical heart sounds with no murmurs, and a normal sinus rhythm on the electrocardiogram.

The patient had no known chronic cardiovascular or systemic diseases; however, his medical history was notable for taking cyproheptadine 4 mg and dexamethasone 0.5 mg since his twenties, following recommendations for weight gain and muscle hypertrophy purposes. Additionally, he had a history of smoking (20 pack-years) and daily use of 10 mg per day methadone. His familial medical history was unremarkable, and he had not reported any known allergies.

**Figure 2:**
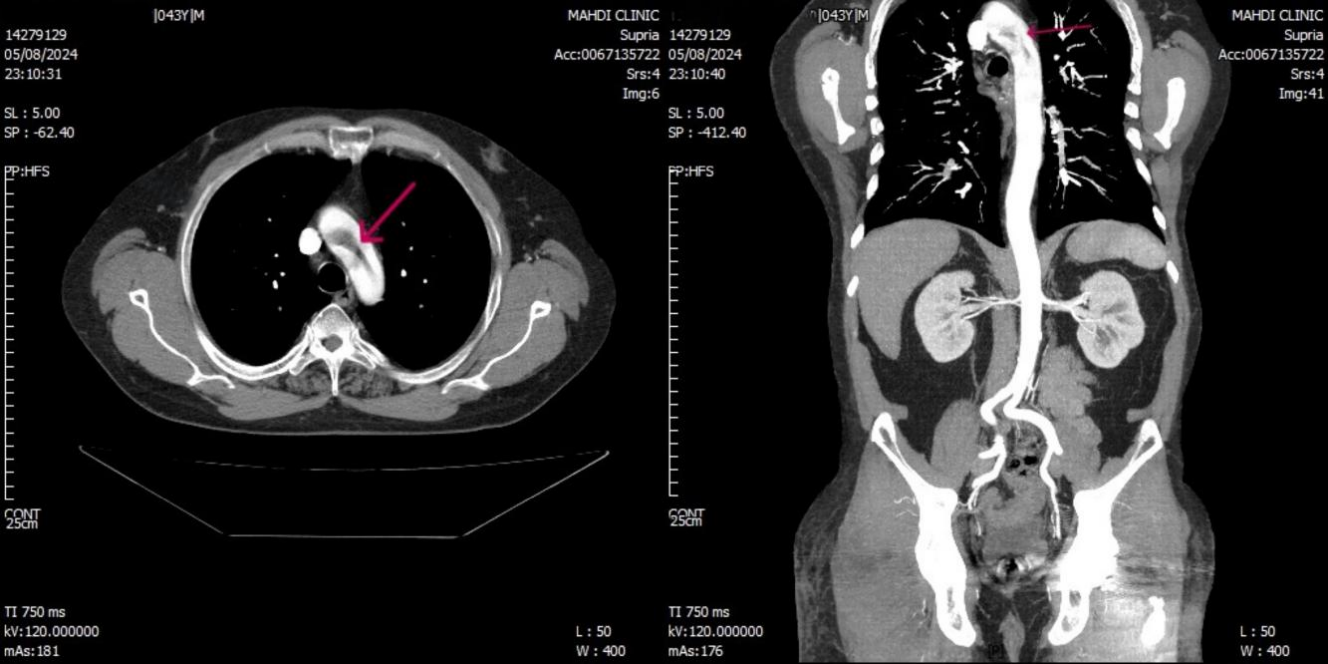
Computed tomography angiography on day 1 demonstrated a large free-floating thrombus within the aortic lumen. The thrombus, measuring 53 mm in craniocaudal length and 13 mm in transverse diameter, appears as a filling defect within the aortic arch and proximal descending aorta (indicated by arrows). It is aligned with the direction of blood flow, with its proximal attachment to the wall of the aortic arch.

**Figure 3:**
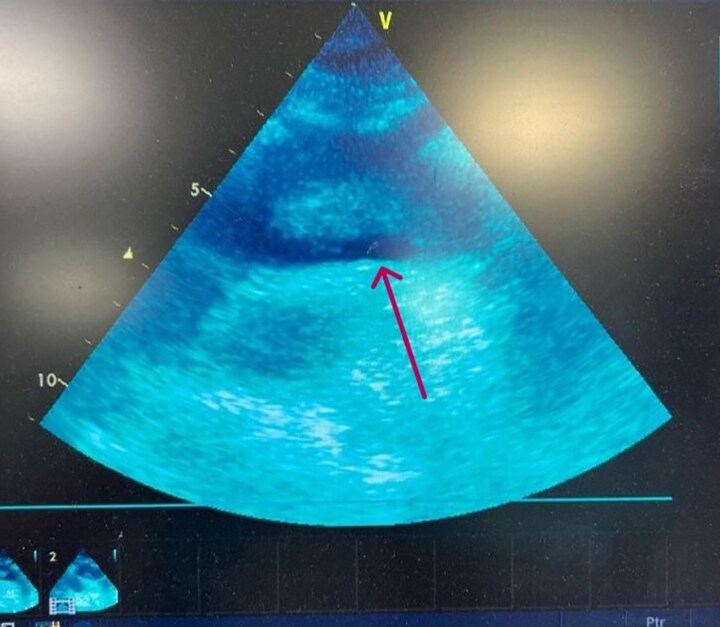
Transoesophageal echocardiography showing a free-floating thrombus within the descending thoracic aorta (indicated by arrow). The thrombus appears as an echogenic mass with a point of attachment to the aortic wall, consistent with findings observed on CTA.

The patient underwent urgent duplex sonography of the left upper limb arteries, revealing thromboembolic occlusions in the left ulnar, radial, and brachial arteries (day 0). An emergency mechanical embolectomy using a Fogarty catheter was performed to relieve the peripheral obstruction (day 0). This procedure effectively restored blood flow to the hand, avoiding amputation. Serial electrocardiography showed no arrhythmias. Further diagnostics aimed to locate the thrombus origin. Transthoracic echocardiography identified mild tricuspid and mitral regurgitation but no structural heart abnormalities (day 2), with a normal ejection fraction of 55%. Computed tomography angiography (CTA) revealed a large, free-floating thrombus in the aortic arch and descending aorta, measuring 53 mm in length and 13 mm in diameter, with no evidence of significant atherosclerotic plaque, ulceration, penetrating aortic ulcer, or aneurysmal change (day 1). The mass was aligned with blood flow, attached proximally to the aortic arch wall, extending into the descending aorta (*Figure 2*). Transoesophageal echocardiography (TEE) further characterized the mass as large, highly mobile, and heterogeneous (day 2) (*Figure 3*). Serological tests for hypercoagulability and vasculitis were negative. No evidence of malignancy, connective tissue disorders, claudication, or family history was found. Ultimately, the diagnosis of acute upper extremity ischaemia caused by a mobile aortic thrombus was confirmed through symptoms, physical exam, imaging, and serology (day 3).

**Figure 4:**
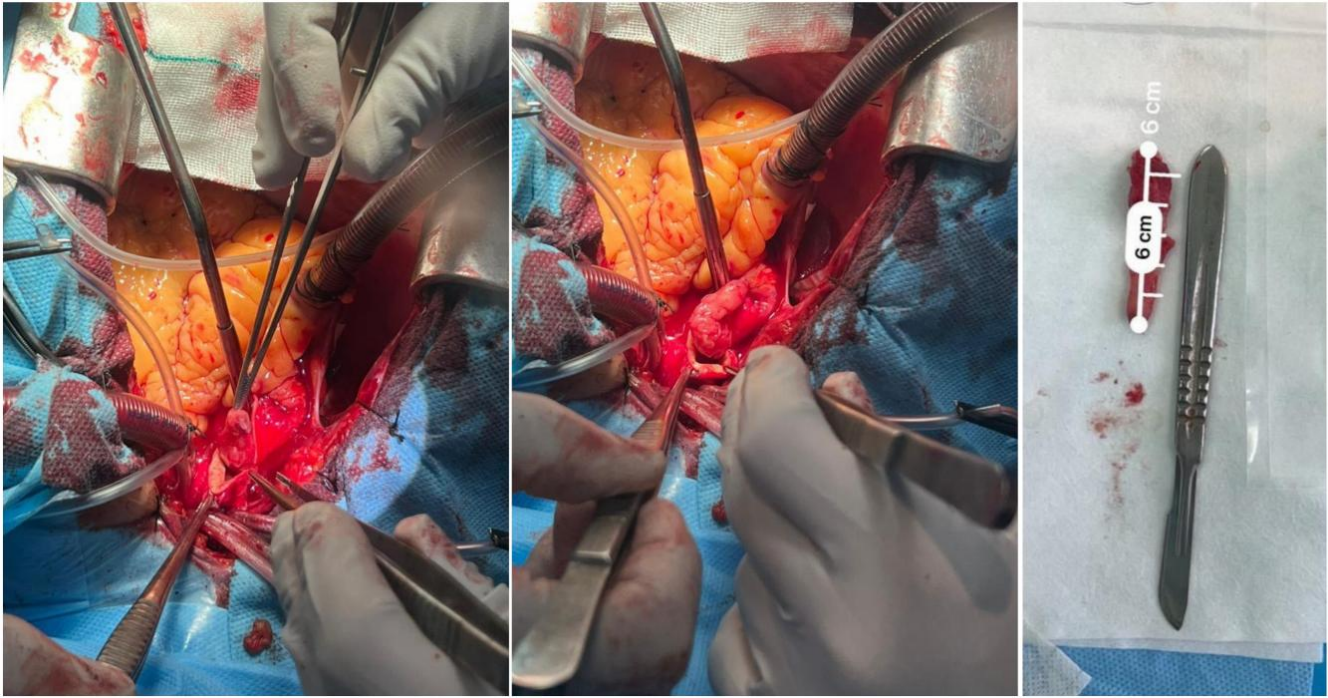
Intraoperative image during open aortic surgery showing the extraction of the free-floating thrombus from the descending thoracic aorta, and gross specimen of the excised thrombus placed beside a scalpel for scale, demonstrating its length of approximately 6 cm. The thrombus appears elongated and intact, consistent with intraoperative findings.

In the case of this young man with a giant, free-floating thrombus in the aorta, our choice of treatment was the surgical excision of the intra-luminal mass, with the primary aim of preventing potential recurrence of peripheral or visceral embolic events. The patient underwent a successful vascular surgery (day 3) procedure to remove a 6 cm thrombus located 2 cm distal to the left subclavian artery (*Figure 4*). Cardiopulmonary bypass (CPB) was established between distal aortic arch cannulation (after origin of left subclavian artery) and RA. Moderate hypothermia was gradually set to 26°C, and the aorta was entered, a tubular mass was excised, and the aortic arch was cleaned. Intraoperative inspection of the aortic wall revealed no significant atherosclerotic plaques or ulcerative lesions at the thrombus attachment site. Aortotomy was closed with Prolene 4-0. Gradually, the patient was rewarmed, and CPB was discontinued after de-airing.

**Figure 5:**
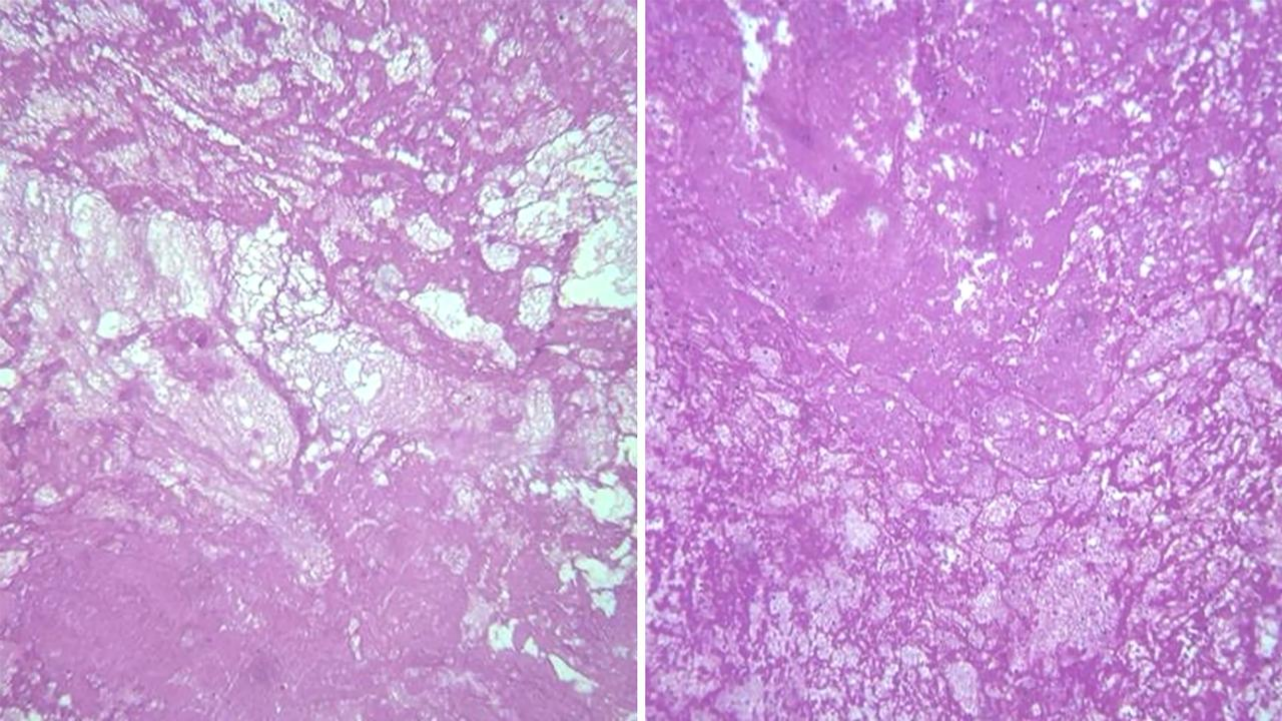
Histopathological section of the excised thrombus (H&E stain) showing fibrin meshwork with entrapped red blood cells, platelets, and scattered leukocytes, consistent with thrombus formation.

Post-operative histopathological analysis of the aortic sample confirmed the thrombotic nature of the mass without features of plaque rupture or underlying atherosclerotic debris, revealing no signs of connective tissue disorders, malignancies, or atherosclerosis (*Figure 5*).

The postoperative course was uneventful. He was set on oral anticoagulation therapy using apixaban (5 mg/BD), with strict advice on smoking cessation and dietary modification. The patient was discharged from the hospital seven days after surgery. At the follow-up assessment after 1, 3, and 6 months, no complications were reported, and 3 months after discharge, follow-up imaging studies confirmed a stable condition without recurrence of aortic thrombus. The patient remained asymptomatic throughout this period, responding well to the prescribed therapeutic regimen.

## Discussion

Aortic mobile thrombi (AMTs) are rare, and the occurrence of a giant floating thrombus in the aorta without severe complications is uncommon. Arterial thromboembolism significantly contributes to morbidity and mortality, yet AMTs are seldom detected before embolic events occur.^2,5^ Although their precise pathogenesis remains unclear, risk factors include immune disorders, intra-aortic atheroma, coagulopathy, structural or functional aortic abnormalities, substance abuse, malignancy, steroid use, and smoking.^2^

Thoracic aortic thrombi, typically associated with atherosclerotic plaque and aneurysms, are more common in the elderly. In younger patients with healthy aortic walls, the presence of an adherent thrombus is extremely unusual and may indicate intrinsic endothelial dysfunction.^1,4,6–9^ Consistent with these observations, our case showed no evidence of atherosclerosis or aneurysm, and structural cardiovascular disease; however, he exhibited several known thrombotic risk factors, such as chronic steroid use, substance dependence, and active smoking.

Steroid use and chronic smoking are recognized contributors to AMT pathogenesis. Corticosteroids can directly injure the endothelium, leading to intimal thickening and promoting thrombosis.^7^ Chronic smoking alters vascular function and substantially increases thromboembolic risk.^1,5^ Consequently, smoking cessation is a critical preventive measure.^1^

CTA plays a central role in diagnosing aortic thrombi and detecting asymptomatic peripheral or visceral emboli. Transthoracic and TEE offer detailed evaluation of thrombus morphology and the proximal ascending aorta, but have reduced utility for distal ascending aorta, aortic arch, or inaccessible abdominal aorta thrombi. Thus, optimal assessment often requires combined imaging modalities: TEE for the cardiac and thoracic aorta, and CT or MRI for the abdominal aorta.^2,9^

There is no established consensus on AMT management. Therapeutic approaches include conservative medical therapy, endovascular intervention, surgical excision, or hybrid techniques.^5,9^ Management decisions depend on thrombus size and mobility, patient comorbidities, clinical presentation, and predisposing risk factors.^5^ Conservative therapy with systemic anticoagulation is a common initial approach, especially in haemodynamically stable or high-risk patients. Some reports show complete thrombus resolution with anticoagulation alone, particularly in non-complicated cases. However, about one-third experience persistent thrombus, and 20–50% have recurrent embolic events during treatment.^6^ This highlights the need for careful imaging and readiness to escalate treatment if the thrombus enlarges or does not regress. Large or highly mobile thrombi, especially over 1 cm, pose a significant embolization risk and often require more aggressive treatment. Nearly 25% of patients initially on anticoagulation eventually need surgery due to persistent or recurrent thrombus.^6^ Endovascular treatment offers a minimally invasive option for patients unresponsive to medical therapy but carries the risk of embolization from thrombus fragmentation during stent deployment or guidewire manipulation. Long-term outcomes remain uncertain, emphasizing meticulous procedural control.^2^ Open surgical excision under cardiopulmonary bypass and moderate hypothermia carries inherent risks, including bleeding, neurologic injury, infection, and systemic inflammatory response. Nevertheless, in the presence of a large, mobile aortic thrombus associated with embolic events, surgical removal provides definitive treatment by eliminating both the thrombus and its attachment site, thereby minimizing recurrence risk.^2^

## Lead author biography


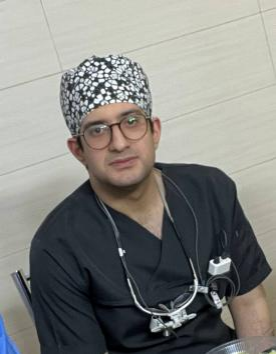


Dr. Mani Moayerifar is a cardiovascular surgery fellow at Tehran University of Medical Sciences. His clinical and research interests focus on advanced surgical techniques in coronary artery bypass grafting, valvular heart disease, and structural heart interventions, including transcatheter approaches to valve repair and replacement. He is committed to improving patient outcomes through evidence-based practice and academic collaboration.

## Data Availability

All data are incorporated into the article.
